# Long term survival following the detection of circulating tumour cells in head and neck squamous cell carcinoma

**DOI:** 10.1186/1471-2407-9-424

**Published:** 2009-12-06

**Authors:** Stuart C Winter, Sally-Anne Stephenson, Selva K Subramaniam, Vinidh Paleri, Kien Ha, Conor Marnane, Suren Krishnan, Guy Rees

**Affiliations:** 1 Department of Otolaryngology, Head and Neck Surgery, Royal Adelaide Hospital, North Terrace, Adelaide, South Australia, 5000, Australia; 2 Institute of Health and Biomedical Innovation, Queensland University of Technology, Kelvin Grove, Queensland, 4059, Australia; 3 Department of ENT, Head and Neck Surgery, Sarawak General Hospital, Kuching, Sarawak, Malaysia; 4 Department of Otolaryngology, Head and Neck Surgery, Freeman Hospital, Newcastle Upon Tyne, UK; 5 Department of Otolaryngology, Head and Neck Surgery. Swansea Hospital, Wales, UK

## Abstract

**Background:**

Techniques for detecting circulating tumor cells in the peripheral blood of patients with head and neck cancers may identify individuals likely to benefit from early systemic treatment.

**Methods:**

Reconstruction experiments were used to optimise immunomagnetic enrichment and RT-PCR detection of circulating tumor cells using four markers (*ELF3*, *CK19*, *EGFR *and *EphB4*). This method was then tested in a pilot study using samples from 16 patients with advanced head and neck carcinomas.

**Results:**

Seven patients were positive for circulating tumour cells both prior to and after surgery, 4 patients were positive prior to but not after surgery, 3 patients were positive after but not prior to surgery and 2 patients were negative. Two patients tested positive for circulating cells but there was no other evidence of tumor spread. Given this patient cohort had mostly advanced disease, as expected the detection of circulating tumour cells was not associated with significant differences in overall or disease free survival.

**Conclusion:**

For the first time, we show that almost all patients with advanced head and neck cancers have circulating cells at the time of surgery. The clinical application of techniques for detection of spreading disease, such as the immunomagnetic enrichment RT-PCR analysis used in this study, should be explored further.

## Background

Head and neck cancers include several histologically distinct cancers that originate in the upper airways. Advances in surgery and radiotherapy over the past few decades have resulted in improved loco regional control however this has not translated to an improved overall survival in all studies. This is due in part to the development of second primary tumours and distant metastases, which occur in up to 20% of patients [1]. Current clinical staging systems frequently fail to predict tumour biology and outcome highlighting the need for more sensitive and specific methods for the detection of spreading tumour cells.

Hematogenous spread of tumor cells from a primary tumor can be considered as a crucial step in the metastasis cascade that can lead eventually to the formation of clinically manifest metastases [2]. Although it is likely that only a small percentage of the tumour cells that are shed into the peripheral blood will successfully lodge and continue to proliferate in distant sites, the presence of these cells within the circulation indicates that the disease has progressed to a stage where metastasis is possible. Indeed, the clinical relevance of the use of methods for the detection of tumour cells disseminated *via *peripheral blood has already been demonstrated by several studies of samples from patients with solid epithelial cancers including breast, prostate and colorectal [3,4]. The use of methods for the detection of haematogenous spread of tumour cells from head and neck squamous cell carcinomas (HNSCCs) may also prove to be of clinical relevance for patient prognosis, staging and monitoring of therapy.

Successful detection of rare tumour cells in blood samples that may contain up to 10^7 nucleated haematopoietic cells can be achieved by combining pre-analytical enrichment with molecular detection of enriched cells using reverse transcriptase-polymerase chain reaction (RT-PCR) assays [5-7]. The sensitive and specific detection of these enriched cells would benefit from the identification of markers that have high levels of tumour-specific expression, but to date no such tumour-specific transcripts have been identified for any solid epithelial cancer [4]. For this reason, and due to the heterogeneity of marker gene expression profiles in most solid tumours including those of head and neck origins, a panel of markers that show increased expression in tumour cells compared to normal epithelial cells is recommended [3,8,9].

In this study, an assay for the detection of circulating tumor cells (CTCs) originating from head and neck squamous cell carcinomas was developed, using reconstruction experiments combining pre-analytical enrichment of HNSCC cell lines inoculated into peripheral blood from normal individuals *via *BerEP4-conjugated immunomagnetic beads (immunobeads) with subsequent RT-PCR detection of a panel of four markers (Immunobead RT-PCR) [10]. The BerEP4 antibody recognises the EpCam protein expressed on the surface of epithelial cells. The markers used for RT-PCR included epidermal growth factor receptor (*EGFR*), the Eph receptor tyrosine kinase B4 (*EphB4*), cytokeratin 19 (*CK19*) and the *Ets *transcription factor, *Elf3/ESX *and were chosen based on reported expression in epithelial cancers including those originating from head and neck origins. Previous studies performed in our laboratory have reported the use of these markers for the identification of circulating breast cancer cells [10,11]. Optimisation experiments using peripheral blood samples from normal individuals showed that none of these four markers can be detected in normal mononuclear cells under the described conditions and that these markers are therefore specific for the detection of circulating epithelial cells [10-12].

Activation of EGFR signalling cascades are associated with cell proliferation, metastasis, angiogenesis and inhibition of apoptosis and *EGFR *has been reported as over-expressed in 80-100% of HNSCC where it is associated with resistance to treatment and poor clinical outcome [13-15]. *EphB4 *is similarly over-expressed in head and neck cancers (100% of 37 cases examined) and knockout studies using siRNA and antisense both *in vitro *and importantly, *in vivo*, have demonstrated that EphB4 has an essential role in many processes that contribute to cancer cell survival and spread [16,17]. Cytokeratins such as *CK19 *are almost exclusively expressed in epithelial tissues and are commonly used as markers for the detection of disseminated tumour cells of epithelial origin [18-20]. Expression of *CK19 *in particular increases in hyperplastic lesions and continues to be expressed in dysplastic and malignant lesions. More recently, Tao et al (2006) report a real-time RT-PCR assay analysing *CK19 *mRNA level as a sensitive and reliable method for the detection of carcinomatous HNSCC cells in lymph nodes and increased CK19 expression has been reported to be associated with local failure and distant metastasis in HNSCC [21,22]. *Elf3*, or epithelial specific with serine box (*ESX*), is a member of the Ets family of transcription factors and its expression is limited to cells of epithelial origin and often up-regulated in cancer cells [23].

The immunobead RT-PCR assay was then used in a pilot study to screen peripheral blood samples taken from 16 patients with advanced HNSCC both before, and immediately after, surgery for the presence of marker-positive CTCs. The clinical relevance of the detection of CTCs in late stage HNSCC is discussed.

## Methods

### Patient Samples

Sixteen patients (14 males and 2 females) with newly diagnosed advanced head and neck squamous cell carcinomas were enrolled prospectively into the study between February and September of 2005 and were treated with curative intent with primary surgery (Table 1). All patients underwent CT examination of the head, neck, chest and abdomen and were staged following a formal panendoscopy. The decision regarding post-operative radiotherapy (RT) or chemotherapy was made in a multidisciplinary setting following surgery in conjunction with the histology results and the patient's wishes. Patients who had previously been treated for HNSCC or who were to receive RT or chemotherapy prior to surgery were excluded. Clinical data including date of birth, sex, tumour subsite, tumour stage, post-operative RT or chemotherapy, disease recurrence and survival were recorded. For the purpose of the analysis tumours were staged based on the pathological resection specimens according to the AJCC/UICC TNM tumour classification [24]. The median age was 59 years (range 44-83 years) with 1 patient diagnosed as stage 1, 6 patients diagnosed as stage 3 and 9 patients diagnosed as stage 4a. Outcome data included all events occurring immediately after surgery. Peripheral blood was collected into dipotassium EDTA tubes from an arterial line at the commencement and end of surgery. For the reconstruction experiments venous peripheral blood samples were collected from normal individuals, also into dipotassium EDTA tubes. All samples were processed within 2 h of collection. Informed consent was obtained from all patients and normal individuals and the research protocol was approved by the Royal Adelaide Ethics Committee.

**Table 1:** Disease characteristics of the 16 patients with head and neck cancers included in this study.

P#	Cancer Site	T	N	M	Stage	P	V	E	Margin	Diff
1	Rt Hypopharynx	1	2b	0	4a	n	n	n	involved	poor

2	Lt Tongue Base	2	1	0	3	n	Y	n	involved	mod

3	FOM	4	0	0	4a	Y	n	x	x	poor

4	RT Supraglottis	2	2b	0	4a	n	n	Y	clear	poor

5	Rt Tongue Base	1	2b	0	4a	n	n	Y	involved	mod

6	Rt Tongue Base	2	1	0	3	n	Y	n	clear	mod

7	Lt Hypopharynx	4	2c	0	4a	n	n	Y	5 mm	poor

8	Glottic	4	0	0	4a	x	n	x	clear	mod

9	Rt Lat Tongue	1	0	0	1	Y	n	x	10 mm	well

10	Lt Tonsil	3	2b	0	4a	n	n	Y	involved	poor

11	Rt Tonsil	2	2b	0	4a	n	n	Y	clear	mod

12	Glottic	3	0	0	3	x	n	x	15 mm	mod

13	Glottic	3	0	0	3	n	n	x	0 mm	mod

14	Transglottic	4	0	0	4a	x	Y	x	1 mm	Mod

15	Rt Lat Tongue	3	1	0	3	n	n	Y	3 mm	poor

16	Rt Lat Tongue	3	0	0	3	n	n	x	0.6 mm	well

**P#**	**Recurrence**	**Radiation**			**Chemo**	**Outcome**			**Before**	**After**

1	Distant	Y - 66 Gy in 32			n	Alive - metastatic disease			neg	POS

2	Local (tonsil)	Y - 64 Gy in 31			n	Died - disease			POS	neg

3	Local (tongue)	Y - 64 Gy in 32			n	Died - disease			POS	neg

4	Local (Node)	Y - 66 Gy in 32			n	Died - disease			POS	neg

5	Local	Y - 66 Gy in 33			n	Died - metastatic disease			POS	neg

6	n	n			n	Alive - Disease Free			POS	POS

7	Distant	Y - 66 Gy in 32			n	died - metastatic disease			POS	POS

8	n	Y - 66 Gy in 33			n	Alive - Disease Free			POS	POS

9	Distant	Y - 66 Gy in 34			n	died - metastatic disease			POS	POS

10	Distant	Y - 66 Gy in 35			Y	died - metastatic disease			neg	POS

11	n	Y - 66 Gy in 36			Y	Alive - Disease Free			POS	POS

12	n	n			n	died - other			neg	neg

13	Local	Y - 64 Gy in 31			Y	Alive - Disease Free			POS	POS

14	Local	Y - 64 Gy in 31			n	died - metastatic disease			neg	neg

15	Local	n			n	died - metastatic disease			POS	POS

16	n	n			n	Alive - Disease Free			neg	POS

P# denotes patient number. Information includes - Site of original cancer (cancer site); tumour size (T); nodal status (N); evidence of metastasis (M); evidence of perineural invasion (P); evidence of vascular invasion (V); evidence of extracapsular invasion (E); whether the margin was involved with tumour after surgery or clear; differentiation of the tumour (Diff); Site of recurrence (Recurrence); whether (Y), or not (n), radiation was given post-surgery (Radiation) including dosage - Grays (Gy) in fractions; whether (Y), or not (n), chemotherapy was given (Chemo); the outcome at last follow-up (Outcome); and whether the patients were positive (POS) or negative (neg), before and after surgery for at least 2 of the RT-PCR markers. Y denotes yes, n denotes no, x denotes unknown.

### Cell culture

The four HNSCC cell lines used in this study were SCC-9, SCC-25, Detroit 562 and FaDu and were all purchased from the American Type Culture Collection (ATCC, Rockville, MD). Cells were cultured in Dulbecco's Modified Eagle Medium (Invitrogen, Carlsbad, CA), pH 7.4, in 75 cm^2 ^vented tissue culture flasks at 37°C in a 5% CO_2 _environment. The medium was supplemented with 100 U/ml penicillin, 100 μg/ml streptomycin, 160 μg/ml L-glutamine and 10% heat-inactivated foetal bovine serum (JRH Biosciences, Lenexa, KS). Cells were collected at >90% confluency by trypsin digestion and centrifugation for 5 min at 1000 rpm, resuspended in phosphate buffered saline (PBS) and counted using a haemocytometer.

### Comparison of attachment of BerEP4 and EMA labelled immunobeads to tumour cells *in vitro*

Dynabeads M-450 covalently coated with sheep anti-mouse IgG (Invitrogen) were labelled with 2 μg of either the BerEP4 antibody (Dako, Denmark A/S) or an EMA antibody (Dako) per mg beads according to the manufacturer's recommendations. In separate experiments, 1 × 10^4 ^cells of all four HNSCC cell lines were resuspended in 1.5 ml of fresh culture medium in 2 ml eppendorf tubes. To this, 5 μl of labeled beads (approximately 2 × 10^5 ^in total) were added and the samples placed on a low speed rotating wheel at 4°C for 1 h. Cells and beads were collected by centrifugation at 1000 rpm for 5 min and the pelleted cells resuspended in 100 μl PBS. A 50 μl sample was placed on a microscope slide and visualised using a Nikon Eclipse E800 light microscope to compare attachment of beads to cells. Cells within several fields of view were counted, the number with beads attached or not recorded, and the number of beads actually attached to cells also counted and recorded. An image of a representative field of view was also taken.

### RT-PCR analysis of marker gene expression in selected HNSCC cell lines

Total RNA was isolated directly from monolayers of HNSCC cell lines FaDu and Detroit 562 using Trizol™ and the manufacturer's recommendations (Invitrogen). A 2 μg sample was then reverse transcribed at 37°C using 3 μl pD(N)_6 _primers (Invitrogen), 200 μM each deoxyribonucleotide triphosphate (dNTP) (Pharmacia, Uppsala, Sweden) and 200 U MMLV reverse transcriptase (Invitrogen) in a reaction volume of 30 μl. Primers specific to the chosen marker genes were as described in Raynor et al, 2002 (Table 2) [10]. The primers used to amplify CK19 have been validated by both Eaton et al (1997) [5] and Hardingham et al (2000) [25] and will not amplify either pseudogenes or genomic DNA at the 68°C annealing temperature used here. Primer pairs were used in PCR reactions performed in a Perkin Elmer PTC100 thermocycler with the following conditions: 2 μl cDNA template, 1.5 mM MgCl_2_, 200 μM each dNTP, 50 ng of each forward and reverse primer, and 0.5 units of Hot Star Taq polymerase in 1× PCR buffer (Qiagen). Cycling conditions included an initial denaturation at 95°C for 15 min, followed by 40 cycles of 96°C for 30 sec, 68°C for 1 min, and 72°C for 1 min, with a final extension of 72°C for 7 min. Amplification products were visualised by ethidium bromide staining following separation by electrophoresis through 1.5% agarose gels.

**Table 2:** Primer pairs for amplification of marker gene sequences for RT-PCR expression and immunobead RT-PCR analyses.

Primer Name	Primer Sequence 5'-3'	Annealing Temperature	Product Size
*ELF3 *F	CTCGGAGCTCCCACTCCTCAGA	68°C	188 bp
		
*ELF3 *R	GCTCTTCTTGCCCTCGAGACAGT		

*EphB4 *F	CCCCAGGGAAGAAGGAGAGCTG	68°C	251 bp
		
*EphB4 *R	GCCCACGAGCTGGATGACTGTG		

*EGFR *F	TGTGAGGTGGTCCTTGGGAATTTGG	66°C	339 bp
		
*EGFR *R	TGCTGACTATGTCCCGCCACTGGA		

*CEA *F	GGTTGGGGTTGCTCTGATATAGCAGC	68°C	97 bp
		
*CEA *R	GCTGTTGCAAATGCTTTAAGGAAGAAGC		

*CK19 *F	GACTACAGCCACTACTACACGACC	68°C	743 bp
		
*CK19 *R	AGCCGCGACTTGATGTCCATGAGCC		

*EpCam *F	GGACCTGACAGTAAATGGGGAAC	68°C	186 bp
		
*EpCam *R	CTCTTCTTTCTGGAAATAACCAGCAC		

The sequences of forward (F) and reverse (R) primers for each of the markers are shown in 5' to 3' direction and were used at the indicated annealing temperature to generate a PCR product of the size indicated in base pairs (bp).

### Reconstruction "spiking" experiments to determine limit of detection of HNSCC cells in blood samples using immunobead RT-PCR

HNSCC cell lines Detroit and FaDu were diluted in PBS and counted and seeded into 10 ml peripheral venous blood samples collected from normal healthy individuals at final concentrations of 10, 50, 100 and 500 cells per 10 ml in triplicate. A recent study by Balasubramanian et al (2009) determined that the number of circulating tumour cells in head and neck patients ranged from 0 to 214 per ml therefore the range we have chosen for these reconstruction experiments will be suitable to determine the lower limit of detection of HNSCC [26].

A 10 μl aliquot of CELLection Epithelial Enrich Dynabeads (Dynal, Invitrogen) was added to each sample before these were placed on a low speed rotating wheel at 4°C for 1 h. Enrichment of the cells to which beads had attached in this time was achieved by juxtaposition to an MPC magnet (Dynal, Invitrogen) for 5 min. The medium and cells to which beads had not attached were removed while the tube was in the magnetic holder and replaced with phosphate buffered saline (PBS). The samples were removed from the magnet and resuspended fully in the PBS wash before a second magnetic collection. Samples were washed in this manner a total of three times to ensure the collection only of cells to which beads had attached. After the final PBS wash, cells were collected to the bottom of the tube and the PBS removed before the addition of 9.5 μl of a cell lysis solution containing 0.3% v/v Nonidet P-40 detergent (Sigma), 500 ng pD(N)_6 _primers (Invitrogen), 20 U of RNasin (Promega, Madison, WI) and 10 mM DTT and this was stored at -80°C until needed. Samples of the lysis buffer only were included as a negative control for the RT-PCR and sample of lysis buffer supplemented with total RNA extracted from HNSCC cell line monolayers were used as a positive control for the RT-PCR. Samples were thawed on ice and the RNA template denatured by incubation at 70°C for 3 min before reverse transcription at 42°C using 3 μl pD(N)_6 _primers (Invitrogen), 200 μM each deoxyribonucleotide triphosphate (dNTP) (Pharmacia, Uppsala, Sweden) and 200 U Superscript III reverse transcriptase (Invitrogen) in a reaction volume of 30 μl. PCR amplification of the chosen marker genes was performed as before using 4 μl of the resultant cDNA sample and 50 cycles of the amplification reaction in a Corbett Rotor Gene 3000. Peripheral blood samples from normal individuals and normal samples spiked with known numbers of tumour cell lines were used as negative and positive controls, respectively.

### Immunobead RT-PCR detection of circulating epithelial cells from patients with HNSCC

Two 10 ml samples of peripheral blood were collected into EDTA tubes from an arterial line at the commencement of surgery and another 2 samples were collected at the end of surgery from the same source. The samples were stored on ice and sent to the laboratory for processing within the same day. The immunobead RT-PCR technique was performed as described above using the beads commercially labelled with the BerEP4 antibody, CELLection Epithelial Enrich Dynabeads (Dynal, Invitrogen), to ensure optimal isolation of circulating HNSCC expressing the EpCam protein. Each patient sample was incubated with 4 million beads on a low speed rotating wheel at 4°C for between 1 and 4 h and then processed for RT-PCR using the method described above.

### Statistics

Statistical analysis and graphs were generated using GraphPad Prism (v 4.0). Loco regional control and survival was analysed using the Kaplan-Meier method and prognostic factors were assessed by Log-rank analysis. Correlations between continuous variables were obtained by linear regression analysis and Spearman's rank correlation. Univariate analyses were performed and a Cox proportional hazard model was used to analyse the effects of patient, tumour and CTCs on disease specific survival, and disease free survival. The following variables were considered in the Cox proportional hazard model - Age, Sex, T stage, N stage and antigen expression. Two-tailed p-values are given and considered statistically significant when p < 0.05.

## Results

### Comparison of attachment of immunobeads labelled with BerEP4 and EMA antibodies to HNSCC cell

**Figure 1:**
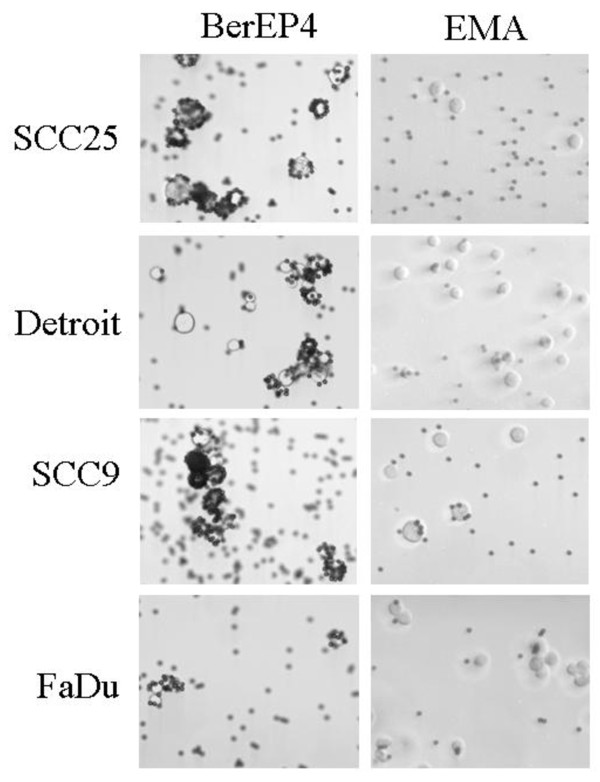
**Comparison of attachment of immunomagnetic beads conjugated with either BerEP4 or EMA antibodies to head and neck squamous cell carcinoma cell lines**. Detroit 562, FaDu, SCC-9 and SCC-15 cells were resuspended in phosphate buffered saline and incubated with labelled beads for 1 h before samples were viewed using a light microscope. The number of cells with beads attached and the number of beads attached per cell were counted by eye at 10× magnification.

Immunomagnetic enrichment of target cells relies on the specific and efficient attachment of the beads to the target cells. Immunomagnetic beads were coated with antibodies targeting two different proteins reported to be present on the surface of tumour cells of epithelial origin, BerEP4 which recognises the EpCam glycoprotein and EMA which recognises the MUC1 protein. After 1 h incubation in the conditions that would later be used to isolate CTCs from patient samples, all cells and beads were collected and attachment of the beads coated with the two different antibodies to 4 different HNSCC cell line cells was determined using light microscopy (Figure 1). Beads coated with the BerEP4 antibody were attached to 98 - 100% of cells from all cell lines, and in most cases cells were heavily rosetted with beads (Table 3). In contrast, beads coated with the EMA antibody attached sparingly to 60% of cells from the SCC-9, 51% of the FaDu cells, 30% of the SCC-25 cells and only 14% of the Detroit cells (Table 3), with only one or two beads attached in most cases. As beads coated with the BerEP4 antibody attached in higher numbers to more cancer cells these were chosen for use in the optimisation of HNSCC cell enrichment method.

**Table 3:** Comparison of the attachment of immunobeads coated with antibodies BerEP4 and EMA to cells from four HNSCC cell lines.

	BerEP4		EMA	
**Cell line**	**Cells with beads attached (%)**	**Average number of beads attached/cell**	**Cells with beads attached (%)**	**Average number of beads attached/cell**

FaDu	100 ± 0	6.4 ± 3.1	51.0 ± 5.0	2.1 ± 1.3

Detroit	98 ± 2	7.0 ± 3.9	13.7 ± 4.2	1.4 ± 0.7

SCC-9	100 ± 0	10.0 ± 2.0	61.5 ± 2.5	2.4 ± 1.1

SCC-25	100 ± 0	13.0 ± 4.0	30.0 ± 2.0	1.4 ± 0.5

Cells were incubated with either BerEP4- or EMA-conjugated beads and a sample viewed using a light microscope. The BerEP4 antibody recognises the EpCam protein and the EMA antibody recognises Muc1/EMA/Mucin 1. The total number of cells and the number of these cells with beads attached were counted and the results are presented as a percentage of cells with beads attached. The average number of beads attached per cell was also determined.

### Expression of markers in HNSCC cell lines

**Figure 2:**
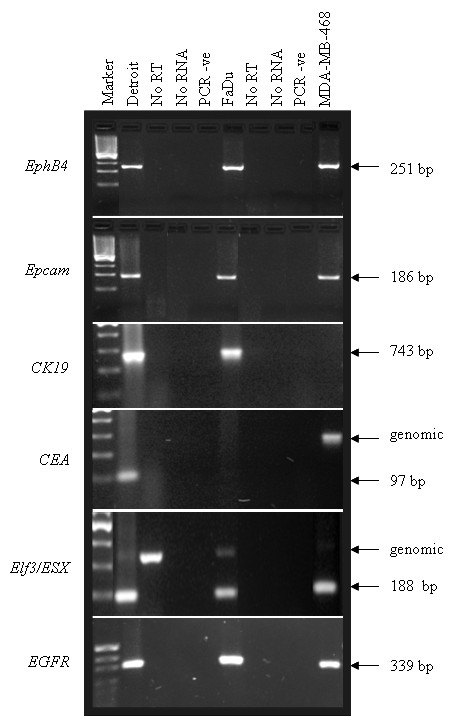
**RT-PCR analysis of the expression of 6 markers in Detroit 562 and FaDu cells**. Total RNA from the Detroit 562 and FaDu cell lines was reverse transcribed then used in individual reactions to amplify products corresponding to each marker as indicated. cDNA from the breast cancer cell line MDA-MB-468 was used as a positive control for *EphB4*, *EGFR*, *EpCam *and *Elf3*. No RT = negative control for the reverse transcription reaction to which no enzyme was added, No RNA = negative control for the reverse transcription reaction to which no RNA was added, PCR -ve = PCR reagent only negative control. Marker = *pUC*19/*Hpa*II. The size of the amplified product is shown in base pairs (bp). A genomic band is seen above the indicated RT-PCR band for *ELF3 *and *CEA*.

The expression of each of the chosen RT-PCR markers was assessed in the representative HNSCC cell lines FaDu and Detroit 562 to determine their suitability as markers for use in the Immunobead RT-PCR assay. Total RNA was extracted from each cell line, reverse transcribed into cDNA, then amplified using primers specific for each of the different markers (Table 2). Transcripts corresponding to *EpCam *were amplified in both of the cell lines, again validating the use of antibodies that target this protein as the antibody label for the magnetic isolation of the head and neck carcinoma cells and confirming the immunofluorescence result that showed bright staining in these lines with the BerEP4 antibody (Figure 2, panel 2). *EphB4*, *EGFR *and *CK19 *were amplified in both cell lines at consistently high levels (Figure 2, panels 1, 3 and 6). *ELF3/ESX *expression was also detectable in both cell lines (Figure 2, panel 5). *CEA *was only amplified from the Detroit 562 cell line and for this reason *CEA *was not chosen as a marker in this study (Figure 2, panel 4).

### Reconstruction "spiking" experiments to determine limit of detection of HNSCC cells in blood samples using immunobead-enrichment coupled with RT-PCR

To determine the limit of detection of HNSCC cells using immunobead enrichment, known numbers of FaDu or Detroit cells (10 to 500) were spiked into 10 ml normal peripheral blood samples in triplicate and the immunobead RT-PCR method was then used to re-isolate and identify them. Cells at the lowest dilution, equivalent to 1 cell per ml of peripheral blood, were detected using the *CK19 *and *Elf3/ESX *markers although not in all samples indicating there may have been some loss of cells during the immunobead isolation or that not all cells express these markers at a sufficient level (Table 4). The lowest level of detection of both cell lines using the *EGFR *and *EphB4 *markers was 5 cells per ml of peripheral blood. Alternatively, failure to consistently detect expression of all markers in samples with low numbers of cells possibly reflects heterogeneity of gene expression in the captured cells.

**Table 4:** Results of reconstruction experiments to optimise the immunobead RT-PCR method.

Marker	Number of cells	FaDu	DETROIT
			

***ESX***	10	1	2

	50	2	3

	100	3	3

	500	3	3

			

***CK19***	10	2	2

	50	3	2

	100	2	3

	500	3	3

			

***EGFR***	10	0	0

	50	3	2

	100	2	2

	500	3	2

			

***EphB4***	10	0	0

	50	2	2

	100	3	1

	500	3	2

Frequency of detection of known numbers of FaDu and Detroit cells (Number of cells) inoculated into normal peripheral blood samples using the immunobead RT-PCR method with specific markers *ESX*, *CK19*, *EGFR *and *EphB4*. Triplicate samples were processed and the number of samples positive with each marker in the two different cell lines is indicated.

### Immunobead RT-PCR analysis of blood samples from patients undergoing surgery for head and neck squamous cell carcinoma obtained prior to surgery

The expression of each of the 4 RT-PCR markers was assessed in duplicate blood samples obtained prior to surgery from 16 patients with head and neck cancers. Aliquots of 500 cells from either the FaDu or Detroit HNSCC cell lines, directly lysed in lysis buffer and reverse transcribed concurrently with the patient samples, were used as positive controls for each RT-PCR assay. As validated by Brakenhoff et al (1999), patients were considered positive for CTCs before surgery if the expression of at least two markers was seen in at least one of the duplicate samples (Table 5) [27]. Using this assay, 11 of the 16 cases analysed were positive for at least 2 markers (68.75%). Of the positive cases, 1/11 (9%) showed expression of all 4 markers, 6 (54.5%) were positive for 3 markers, and 4 (36%) were positive for 2 markers. Importantly, two of the patients in which CTCs were detected prior to surgery had no other evidence of spreading disease (no perineural invasion, vascular invasion or extracapsular spread). Duplicate samples were analysed for agreement using a 2 tailed paired t-test. There was a significant correlation between the samples taken pre-operatively for the antigen ESX only, (p = 0.02).

**Table 5:** Results of Immunobead RT-PCR screening of peripheral blood samples from the 16 patients with head and neck cancers included in this study for marker expression.

	Marker 1				Marker 2			
	
	*ESX*	*ESX*	*ESX*	*ESX*	*EGFR*	*EGFR*	*EGFR*	*EGFR*
**P#**	**BF 1**	**BF 2**	**AF 1**	**AF 2**	**BF 1**	**BF 2**	**AF 1**	**AF 2**

1				POS				

2								

3	POS							

4	POS					POS	POS	

5	POS							

6	POS	POS		POS			POS	POS

7	POS	POS	POS	POS			POS	POS

8	POS	POS		POS	POS	POS	POS	

9						POS	POS	POS

10								POS

11	POS	POS			POS		POS	POS

12	POS							

13			POS	POS	POS	POS		

14								

15	POS				POS		POS	

16							POS	

								
						
	**Marker 3**				**Marker 4**			
	
	***EphB4***	***EphB4***	***EphB4***	***EphB4***	***CK19***	***CK19***	***CK19***	***CK19***

**P#**	**BF 1**	**BF 2**	**AF 1**	**AF 2**	**BF 1**	**BF 2**	**AF 1**	**AF 2**

1						POS	POS	

2	POS				POS	POS		

3			POS		POS	POS	POS	

4				POS		POS		

5					POS	POS		POS

6	POS	POS		POS	POS	POS	POS	POS

7		POS	POS	POS	POS	POS	POS	

8	POS	POS		POS	POS	POS	POS	POS

9	POS	POS			POS	POS	POS	POS

10			POS					POS

11	POS						POS	POS

12								POS

13	POS	POS		POS	POS	POS	POS	

14								

15		POS	POS			POS	POS	

16					POS	POS		POS

P# denotes patient number. Duplicate samples taken before surgery (BF) or after surgery (AF) were screened for expression of the four markers *ESX*, *EGFR*, *EphB4 *and *CK19*. Samples that were positive for individual markers are indicated (POS).

### Immunobead RT-PCR analysis of blood samples from patients undergoing surgery for head and neck squamous cell carcinoma obtained after surgery

The expression of each of the 4 RT-PCR markers was also assessed in duplicate blood samples obtained after surgery from the same cohort of patients. Patients were again considered positive if the expression of at least two markers in at least one of the duplicate samples was detected after surgery (Table 5). Ten of the 16 cases analysed were positive for at least 2 markers (62.5%). Of the positive cases, 3/10 (30%) showed expression of all 4 markers, 4 (40%) were positive for 3 markers, and 3 (30%) were positive for 2 markers.

### Expression of RT-PCR markers by stage

The expression of RT-PCR markers was evaluated against clinical stage for all 16 patient samples of known stage. Positive RT-PCR marker expression was detected before surgery in 1/1 (100%) patients with Stage 1 disease, 4/6 (67%) patients with Stage 3 disease and 6/9 (67%) patients with Stage 4a disease. Positive RT-PCR marker expression was detected after surgery in 1/1(100%) patients with Stage 1 disease, 5/6 (83%) patients with Stage 3 disease and 4/9 (44%) patients with Stage 4a disease. Association of marker expression with stage was tested using Fisher's exact test. The detection of disseminated disease using RT-PCR markers was not significantly associated with tumour stage.

### Expression of RT-PCR markers by tumour histology

Of the 16 patients, 2 were classified as well differentiated tumours, 8 patients had moderately differentiated tumours and 6 had poorly differentiated tumours. One of the 2 patients with well differentiated tumours (50%), 6/8 patients with moderately differentiated tumours (75%) and 4/6 patients with poorly differentiated tumours (67%) were positive for at least two markers before surgery. Both of the 2 patients with well differentiated tumours (100%), 4/8 patients with moderately differentiated tumours (50%) and 4/6 patients with poorly differentiated tumours (67%) were positive for at least two markers after surgery.

### Comparison of individual marker expression prior to and after surgery

There was marked variability in expression of each marker in samples collected prior to and after surgery probably reflecting likely genetic heterogeneity of the tumour cells enriched from different individuals. Not all patients who were positive before surgery were also positive after surgery and *vice versa*. Furthermore often only one of the two samples collected either prior to or after surgery was positive. The results are summarised in Table 5.

### Comparison of patient outcome and marker expression, case-by-case

Four patients (Patients 6, 8, 11 and 16) had no evidence of local recurrence or metastatic disease at the most recent follow-up (22 - 33 months after surgery) even though three of these patients were positive for at least 2 markers prior to surgery and all four were positive for at least 2 markers after surgery. Only one patient (Patient 14) tested negative for marker expression in all samples tested. This patient did however have evidence of lymphovascular invasion and local recurrence and later died with metastatic disease.

Six other patients (Patients 2, 3, 4, 5, 13 and 15) had a local recurrence of disease. For two of these patients (Patients 2 and 5) the margins were involved after surgery and there was evidence of either lymphovascular (Patient 2) or extracapsular spread (Patient 5) on pathological examination. Both of these patients were also positive for 2 markers prior to their surgery but CTCs were not detected in peripheral blood samples taken immediately after. The other four patients with local recurrence were also positive for at least two markers prior to surgery and all remained positive after surgery. Three patients also all had evidence of spread at the time of surgery, either perineural (Patient 3), or extracapsular (Patients 4, 15). Only one of these six patients was alive and disease free at the last follow-up, a year after having had a second surgery to remove the local recurrence (total laryngectomy). Although pre- and post-operative blood samples were not taken during this second surgical event, both duplicate peripheral blood samples taken from this patient prior to the first surgery were positive for *EGFR*, *EphB4 *and *CK19 *indicating that CTCs were already in the peripheral blood of this patient.

Four patients (Patients 1, 7, 9 and 10) relapsed with metastatic disease at a distant site. Patients 7 and 9 were positive for at least two markers prior to surgery and remained positive after surgery suggesting that the diseases in these patients had already begun the metastatic process prior to surgery. Both of these patients also had evidence of spread, either extracapsular (Patient 7) or perineural (Patient 9), as determined by pathological examination. Patient 1 was positive for both *ESX *and *CK19 *in one of the two peripheral blood samples taken after surgery. Similarly, Patient 10 was negative for all markers prior to surgery but was positive for *EGFR*, *EphB4 *and *CK19 *after. Both patients were later diagnosed with distant metastases and died from their diseases 33 and 19 months after the initial surgery, respectively. This may suggest that tumor cells were shed during surgical manipulation of the tumour, however the surgical margin was involved with tumour in both cases and there was evidence of extracapsular spread in Patient 10.

Patient 12 was not included in this analysis due to death from causes unrelated to the disease or surgery.

### Survival analysis

**Figure 3:**
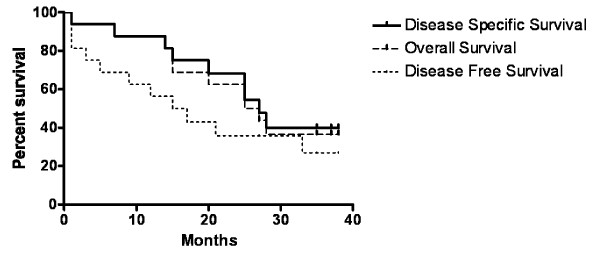
**Kaplan-Meier survival analysis**. Comparison of survival data from patients positive or negative for epithelial cells in blood.

Kaplan-Meier survival analysis was performed using recurrence or death from disease as endpoints. The 3 year actuarial overall survival for the cohort was 37%, disease specific survival was 40%, and disease free survival was 27% (Figure 3). CTCs expressing each antigen from samples taken both pre- and post-operatively were compared to overall survival, disease specific survival and disease free survival but were not associated with significant differences in survival or disease free survival in the cohort. When considering the 6 patients (Patients 6, 7, 8, 11, 13 and 15) who were positive for the three markers, (ESX, EGFR and EphB4) there was no significant correlation with overall survival, p = 0.22).

## Discussion

Despite significant advances in imaging, surgical management and therapeutic options for the treatment of cancers in general, there have been only modest improvement in the outcomes for patients diagnosed with cancers of the head and neck. The development of molecular marker-based strategies have the potential to provide further information that can contribute to the prognosis, treatment selection and surveillance for recurrence and secondary tumour development [4]. In particular, the identification of CTCs within peripheral blood samples and bone marrow is an emerging area of research that may facilitate the identification of tumours with the potential of early metastasis [28].

Highly sensitive and specific techniques are required to distinguish tumour cells from haematopoietic cells in peripheral blood and bone marrow samples when they are present in low concentrations, often as few as one to five tumour cells per 10^6 to 10^7 haematopoietic cells [4,26]. A variety of techniques have been employed to identify these tumour cells including immunocytochemistry, typically using antibodies to detect cytokeratins, and RT-PCR analysis using total RNA extracted from the nucleated cell fraction in whole blood and lineage specific markers [8,29-32]. More recently, methods for the positive or negative enrichment of tumour cells, prior to detection using PCR and RT-PCR assays, have reduced the rate of false positivity from illegitimate expression of some more commonly used markers in normal blood cells [33-35].

In this study, the immunobead RT-PCR method, combining pre-analytical enrichment with magnetic beads coated with the EpCam-specific BerEP4 antibody with RT-PCR amplification of a panel of four markers, was optimised for the detection of circulating HNSCC tumour cells in peripheral blood samples. As it was considered unlikely that normal epithelial cells would be present within the peripheral blood [35,36], and malignant cells often continue to express markers that are characteristic of or specific for the normal tissue from which they originate [35], we used a combination of markers that were either expressed specifically in epithelial cells (*Elf3*/*ESX *and *CK19*) [19-24] or have been reported to be over-expressed in a high percentage of tumour cells from head and neck origins (*EGFR *and *EphB4*) [14-18]. These markers were also chosen for this study as they have already proven useful in other immunobead RT-PCR studies using peripheral blood samples from patients with breast and colon cancer [10,11,37]. Reconstruction experiments using known numbers of cells from two HNSCC cell lines, FaDu and Detroit, inoculated into peripheral blood samples of normal individuals, were used to optimise the immunobead RT-PCR method for the detection of head and cell cancer cells and confirmed that this technique was able to consistently identify the presence of tumour cells with the required sensitivity of 1 to 5 cells per ml of peripheral blood [4,26].

The immunobead RT-PCR assay was then used in a pilot study to assess the long-term follow-up of 16 patients with advanced stage HNSCC treated with primary surgical resection and then with or without postoperative chemotherapy and/or radiotherapy. The 5-year actuarial disease free and survival is in line with published data [38]. Not surprisingly, given that all of the patients included in this study were either diagnosed with advanced disease, or had evidence of disease spread at the time of surgery (Patient 9), 14/16 (87.5%) of the patients included in this study did test positive for at least two of the chosen markers either prior to or directly after surgery and are therefore considered to have CTCs within their peripheral blood. The presence of cells expressing these epithelial specific and/or tumour-associated mRNAs at body sites where these transcripts are not normally present, such as peripheral blood, implies that the tumours from these patients have gained the capacity to spread through the vascular system.

Marker positive cells were detected before surgery in two patients, and after surgery in these two and a further two patients who are alive and considered disease free as at the most recent follow-up. Whilst the clinical significance of CTCs in the peripheral blood of patients with HNSCC remains unclear [4,28,39-41], this result would suggest that the tumour cells in these patients have gained access to the vascular circulation and they therefore have an increased risk of relapse and should be carefully monitored for potential recurrence/relapse. In this study three patients, who were not positive for CTCs prior to surgery, tested positive for CTCs after surgery. This may indicate that surgical manipulation of the primary tumours in these individuals has resulted in dissemination of tumour cells into the circulation but the clinical relevance of cells disseminated by surgery is a subject of debate.

Only one patient (Patient 14) was not identified as having CTCs either before or after surgery. This patient did however have evidence of lymphovascular invasion and later died from a locoregional recurrence and metastatic disease. It is well recognised that CTCs are not always detected even when advanced metastatic disease is present and this is presumed to be the result of either sampling errors [42], intermittent tumor cell shedding [43], effective immune surveillance [44] or stochastic effects at the lower limits of sensitivity of the assay [45]. In addition, it cannot be assumed that the assay used in this study was able to detect all CTCs with uniform sensitivity even though a panel of four different markers was used, particularly if the tumour cells from this patient did not actually express these markers. Possible improvements might be gained through the inclusion of further markers to ensure the identification of all CTCs. Promising markers for head and neck cancers include parathyroid hormone-like hormone (*PTHLH*, also known as *PTHrP*), serpin peptidase inhibitor, clade B (ovalbumin), member 3 (*SERPINB*, also known as *SCCA*), and lymphocyte antigen 6 complex, locus D (LY6D, also known as E48) [8,29,30].

Ten patients included in this study had CTCs positive for *EGFR*. *EGFR *is commonly up-regulated in head and neck cancers and this receptor has emerged as a key molecular target for anti-cancer therapies including the monoclonal antibody therapy Cetuximab licensed for the treatment of HNSCC [46]. The immunobead RT-PCR method, developed here for detecting CTCs expressing *EGFR*, may provide an important future clinical application for monitoring the efficacy of systemic therapies through the real-time monitoring of EGFR-positive CTCs in cancer patients during their treatment.

Detection of disseminated cells can identify patients who may develop distant metastases and therefore have reduced survival. A recent study comparing immunocytochemistry with a pan-cytokeratin antibody and RT-PCR for *LY-6/E48 *expression for detection of disseminated cells in bone marrow and blood from 40 patients with HNSCC, showed that detection of cells in pre-operative bone marrow and blood correlated with the development of both locoregional recurrence and distant metastasis [30]. In the study reported here however, there was no association between the detection of CTCs and any significant reduction in progression free survival or overall survival. This may be due to both the small number of patients included in this pilot study and the fact that nearly all of them were diagnosed with advanced disease, which is associated with poor prognosis.

## Conclusion

An immunobead RT-PCR method, combining immunomagnetic enrichment of tumor epithelial cells with RT-PCR analysis of *ESX*, *EGFR*, *EphB4 *and *CK19 *was optimised using reconstruction experiments with two HNSCC cell lines, then used to detect CTCs in the peripheral blood from 16 patients with advanced HNSCC. Samples from fourteen of these patients were positive for at least two of the chosen markers and were therefore considered positive for CTCs. This is the first study to report that, at the time of surgery, circulating cells can be detected in the peripheral blood of almost all patients with advanced head and neck cancers. This result demonstrates the clinical utility of immunobead RT-PCR as a minimally invasive technique that facilitates the rapid screening of patient peripheral blood for CTCs associated with head and neck cancers, and therefore the potential for developing this assay for future incorporation into the clinical setting. Techniques that accurately detect CTCs will find utility for detection of spreading disease, routine monitoring of disease progression and for determining the efficacy of adjuvant systemic treatments.

## Competing interests

The authors declare that they have no competing interests.

## Authors' contributions

SW co-ordinated the collection and analysis of the clinical data. SAS, SKS and KH performed the experiments and analysed the data. SW and SAS drafted the manuscript. KH assisted with editing the manuscript. VP, CM and SK provided access to clinical samples and were involved in interpreting the data and editing the manuscript. SW performed the statistical interpretation of the data. GR was responsible for the overall conception and design of the project, for interpretation of the data and editing of the manuscript.

## Pre-publication history

The pre-publication history for this paper can be accessed here:

http://www.biomedcentral.com/1471-2407/9/424/prepub
